# Passive case detection for canine visceral leishmaniasis control in urban Brazil: Determinants of population uptake

**DOI:** 10.1371/journal.pntd.0009818

**Published:** 2021-10-08

**Authors:** João Gabriel G. Luz, Amanda G. de Carvalho, João Victor L. Dias, Luis Claudio L. Marciano, Sake J. de Vlas, Cor Jesus F. Fontes, Luc E. Coffeng

**Affiliations:** 1 Department of Public Health, Erasmus MC, University Medical Center Rotterdam, Rotterdam, The Netherlands; 2 School of Medicine, Institute of Exact and Natural Sciences, Federal University of Rondonópolis, Rondonópolis, Brazil; 3 Post-graduation Program in Health Sciences, Faculty of Medicine, Federal University of Mato Grosso, Cuiabá, Brazil; 4 School of Medicine, Federal University of Jequitinhonha and Mucuri Valleys, Teófilo Otoni, Brazil; University of Iowa, UNITED STATES

## Abstract

**Background:**

In Brazil, the transmission of *Leishmania infantum* in urban settings is closely related to infection among dogs, with occasional transmission to humans. Serological screening of dogs for *Leishmania* spp. infection on requests of their owners (passive case detection) represents a frequent, but little studied, practice within the scope of Brazilian public health. This study identified factors associated with canine visceral leishmaniasis (CVL) diagnosis-seeking behavior of dog owners in Rondonópolis (236,000 inhabitants), a municipality in Central-Western Brazil where VL is endemic. Also, we evaluated the profile of dog owners and their animals screened on free demand.

**Methodology/Principal findings:**

Using mixed effects negative binomial regression, we modelled the number of dogs screened for *Leishmania* infection on free demand per neighborhood from 2011 to 2016 as a function of time-dependent predictors (current or recent canine seropositivity and human VL incidence), distance to the screening site, and demographic variables. We assessed potential delays in the effect of time-dependent predictors on the outcome. Among 12,536 dogs screened for *Leishmania* infection, 64.2% were tested during serosurveys and 35.8% were tested on free demand. Of these, 63.9% were positive. Uptake of screening under free demand was strongly associated with higher levels of canine seropositivity in the neighborhood (current or recent) and decreasing distance to the screening site. A subsample of dog owners (n = 93) who sought CVL screening between 2016 and 2017 were interviewed in more detail. Owners with better socioeconomic status and dogs with apparent CVL clinical manifestations prevailed among them.

**Conclusions/Significance:**

To support timely CVL management, passive case detection along with awareness activities aimed at dog owners should be encouraged in endemic areas. Screening sites should be prioritized in accessible zones, as well as in socio-economically disadvantage areas. In parallel, CVL active case detection should be continued as a surveillance tool to guide control actions.

## Author summary

In Brazil, visceral leishmaniasis (VL) occurs typically in urban settings, where dogs play a role as reservoirs and control efforts have failed to reduce disease occurrence. Active screening for *Leishmania* spp. canine infection by serosurveys is an important pillar of the national VL control program. However, many municipalities also detect cases passively by testing animals on request of individual dog owners. For a VL-endemic municipality in Brazil, we show how the number of dogs tested on free demand in the area increased from 2011 to 2016, with seropositivity consistently exceeding 47%. Increased diagnosis-seeking behavior within neighborhoods was associated with a decrease in the distance to the screening site and higher (recent) levels of canine seropositivity in the neighborhood. The data also suggested that dog owners with better socioeconomic status and animals with clinical signs of canine VL (CVL) predominated among those who sought screening. As passive case detection contributes to timely diagnosis of CVL, it should be encouraged in VL endemic areas. Diagnosis seeking-behavior could be increased by setting up screening sites in multiple accessible areas and especially in areas that are most affected by CVL. In parallel, population-based serosurveys must continue as surveillance tools to guide VL control actions.

## Introduction

Dogs (*Canis familiaris*) are considered the main domestic reservoir hosts in the anthropozoonotic transmission cycle of the parasite *Leishmania (Leishmania) infantum*, which is one of the causative agents of visceral leishmaniasis (VL). *L*. *infantum* is transmitted by the bite of phlebotomine sand flies (Diptera: Psychodidae), and infections occur predominantly in Central Asia, the Middle East, North Africa, the Mediterranean region and Latin America [1]. Within the Americas, more than 90% of all the human cases of VL occur in Brazil [2]. From 2007 to 2014, at least 28,000 human cases and 1,700 human deaths due to VL were recorded nationwide. Most of these records come from medium and large-sized urban centers in the Northeast, Southeast, and Central-Western regions [3]. Regarding canine infection, it has been demonstrated that the circulation of *L*. *infantum* among dogs is usually associated with human VL cases in the country [4–5]. In addition, it is estimated that canine VL (CVL) prevalence, including asymptomatic and symptomatic dogs, ranges from 3.1 to 40% in Brazilian endemic areas [6].

Along with the diagnosis and management of human cases and vector control by residual insecticide spraying, serological screening for *Leishmania* spp. infection in dogs represents one of the central pillars of the Visceral Leishmaniasis Control and Surveillance Program (VLCSP) advocated by the Brazilian Ministry of Health. Thus, serological screening can inform policy for surveillance and control actions against VL. The current recommendation is that seropositive dogs (i.e. positivity for *Leishmania* spp. antibodies in two serial techniques) are euthanized [7].

For both surveillance and control purposes, systematic serosurveys for *Leishmania* spp. screening among dogs are recommended [7]. In addition, some Brazilian public health surveillance units also test animals on request of individual dog owners, allowing for passive case detection through diagnosis-seeking behavior. Although passive detection of CVL is a frequent practice nationwide [8–12], there are no studies specifically addressing why people take up CVL serological screening on free demand. A better understanding of this practice would be useful to guide control actions toward the canine reservoir management in zoonotic VL endemic areas. Therefore, in the present study we attempted to identify factors associated with CVL diagnosis-seeking behavior in the scope of public health in the municipality of Rondonópolis, an area that recently emerged as endemic for VL in Central-Western Brazil. In addition, we analyzed the profiles of dog owners and their animals screened on free demand.

## Methodology

### Ethics statement

Ethical approval for this study was obtained from the Ethical Committee for Human Research of Júlio Müller University Hospital (CAAE N° 52023215.5.0000.5541). Written informed consent was obtained from all interviewed participants.

### Study area

**Fig 1:**
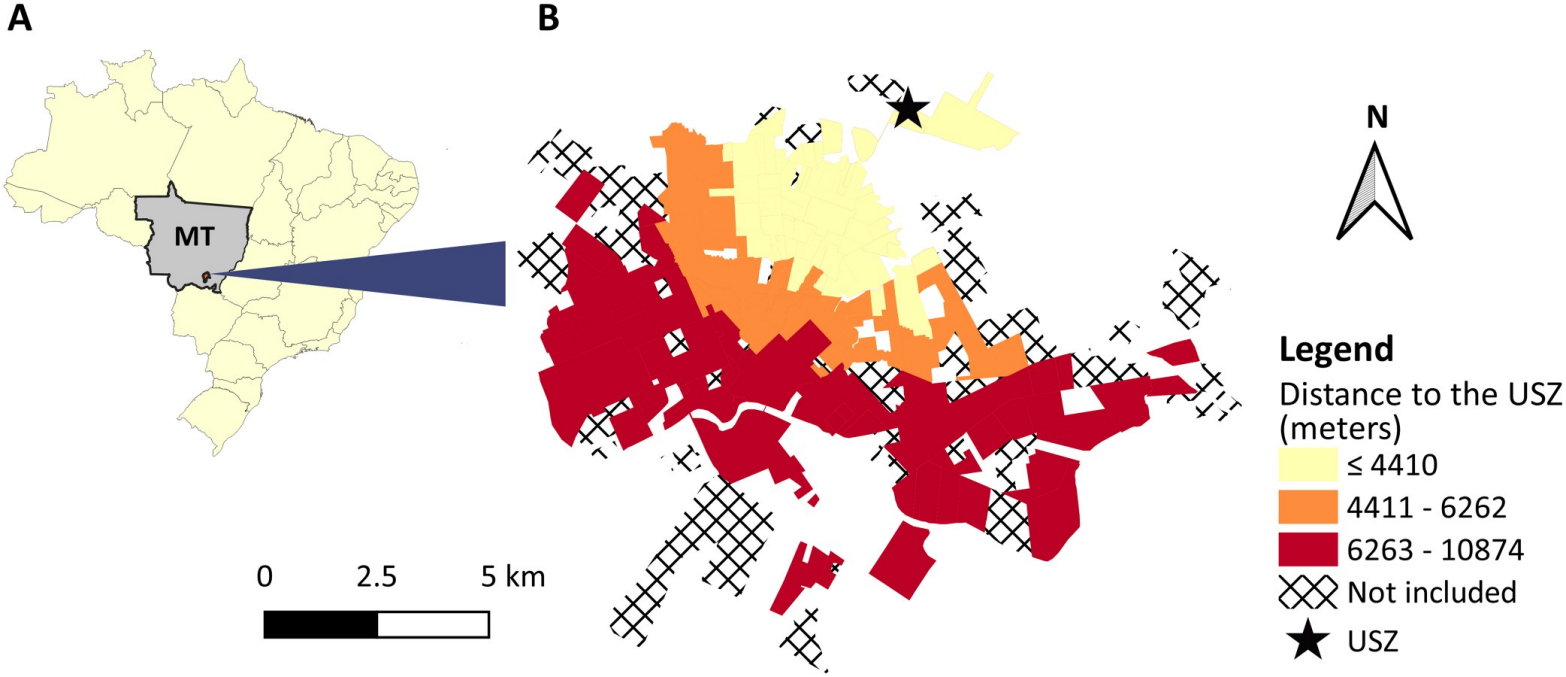
Geographic characterization of the study area. (A) represents the location of the municipality of Rondonópolis in the state of Mato Grosso (MT), Brazil. (B) represents the tertiles of distribution of the linear distance between the geographical coordinates of the centroids of each urban neighborhood included in the present study to the Unit of Surveillance in Zoonosis (USZ). Neighborhoods that were not listed in the last Demographic Census [14] or with human population ≤ 30 inhabitants were excluded from the analysis (black diagonal raster pattern). Digital georeferenced database of the neighborhoods was provided by the Municipal Health Department of Rondonópolis.

The municipality of Rondonópolis is located in the Southeast mesoregion of the state of Mato Grosso, Central-Western Brazil (Fig 1A). It has a surface area of 4,686 km^2^ and a human population currently estimated at 236,042 individuals [13]. According to the last Demographic Census [14], its urban area is composed of 230 neighborhoods.

The first record of autochthonous human VL in Rondonópolis dates from 2003. Since then, 210 autochthonous cases were reported in the municipality; especially as of 2007, when Rondonópolis emerged as the main endemic area of the State [15]. Recently, peaks in incidence (12.6 VL cases/100,000 inhabitants in 2011) and lethality (8.6% in period 2011–2016) have been observed [16]. CVL is highly prevalent in the area; the seroprevalence among domestic dogs in Rondonópolis has been estimated at 19.2%, with seroprevalences reaching up to 35.1% in some areas [17].

Surveillance and control measures for VL are focused on humans, vectors, and reservoirs and are coordinated by the local Municipal Health Department. In particular, the management of canine reservoirs (i.e. active and passive CVL case detection as well as euthanasia procedures) is conducted by the Unit of Surveillance in Zoonosis (USZ) of Rondonópolis, which is located in the extreme north of the urban area of the municipality (Fig 1B). The USZ was the only public screening site in which dog owners could take their animals to be tested free of charges during the study period. Owners could also seek private veterinary service for screening. As of September 2016, miltefosine has officially become available for treatment of CVL in the area [18], but its use has only been scaled up over subsequent years.

### Study design

This was an epidemiological study in two parts. The first part was an ecological study focused on investigating factors associated with CVL diagnosis-seeking behavior at the neighborhood level in the municipality of Rondonópolis, from 2011 to 2016. In the second part, we analyzed the profiles of a convenience sample of dog owners and their respective animals who attended the USZ seeking screening for CVL over the period of 1 year, based on interviews using a semi-structured questionnaire.

### Factors associated with CVL diagnosis-seeking behavior at the neighborhood level

#### Data collection and management

Information on the occurrence of human VL (date of notification and residential address) were collated from the Brazilian Notifiable Diseases Information System, which is coordinated by the Epidemiological Surveillance Sector of the Municipal Health Department of Rondonópolis. We considered all the confirmed cases reported in the urban area of the municipality from 2011 to 2016. Relapses or non-autochthonous cases were excluded. All demographic data were acquired from the last Demographic Census [14] and the last National Health Survey [19]. Case counts were aggregated by neighborhood and 1-month time periods. Neighborhoods that were not listed in the last Demographic Census were excluded from the analysis (black diagonal raster pattern in Fig 1B).

Data on dogs submitted to serological screening for *Leishmania* spp. infection—on free demand or during serosurveys—were extracted from the USZ physical registry books. We included records from domestic animals tested between 2011 and 2016, whose owners resided in the urban area of Rondonópolis. Individuals with unidentified residential addresses were excluded. The following variables were digitized: date of blood collection, residential address, and results of serology. It is noteworthy that between 2011 and 2012, following contemporary guidelines of the Brazilian Ministry of Health, a dog was considered positive for *Leishmania* spp. infection when presented both reactivity in an enzyme-linked immunosorbent assay (ELISA) and immunofluorescence antibody test [7]. From 2013 onwards, the infection was confirmed with dual positivity in a rapid chromatographic immunoassay based on the dual-path platform (TR DPP) and ELISA [20]. The number of dogs tested and the number of seropositive dogs were aggregated by modality (free demand or serosurvey), neighborhood, and 1-month time periods.

#### Estimating dog population size

To calculate trends in the likelihood that dog owners seek out testing for potential CVL, it was necessary to assess the domestic canine population size in each neighborhood of the municipality. This information was not available for Rondonópolis. Therefore, given the well-known relationship between dog ownership and demographic characteristics [21], we set out to infer the number of dogs per neighborhood from demographic data, using a statistical model. We calibrated the statistical model using data from the last National Health Survey for the state of Mato Grosso [19]. Briefly, this survey is a household-based survey conducted nationwide. Data (including the number of domestic dogs and demographic variables) were originally collected from permanent private households (PPHs) randomly selected from primary sampling units (PSUs) in all the Brazilian states [22]. Here, PSUs can be considered comparable to neighborhoods as a geographical unit of aggregation.

Out of the 162 PSUs sampled in the state of Mato Grosso, we only included data from urban PSUs (n = 138). Given that the outcome variable constituted count data that were possibly overdispersed, we modelled the number of domestic dogs in each PSU as a function of a set of candidate demographic predictors using a negative binomial regression. The following predictors were chosen based on their availability for the neighborhoods of Rondonópolis: mean number of inhabitants per PPH, mean monthly nominal income per PPH (in Brazilian minimum wages), proportion of PPHs with literate heads, proportion of PPHs according to the housing type (house or apartment), proportion of PPHs with public water supply, proportion of PPHs with public garbage collection, and proportion of PPHs with sewage system. In addition, we included the natural logarithm of the number of PPHs in each PSU as an offset term, such that the exponents of the model coefficients represent the relative difference in number of domestic dogs per PPH.

For negative binomial modelling, we selected all the variables with a *p*-value < 0.20 in univariate analysis that were not highly correlated with other predictors. The final model was developed by a stepwise forward procedure. We used the Akaike Information Criterion to assess if the addition of predictors improved the model fit. Variables with a *p*-value < 0.05 were maintained in the final model, as well as those that improved the model fit and were considered important according to the literature. The model assumption of overdispersion was checked using a likelihood ratio test by comparing the final negative binomial model with a Poisson model containing the same explanatory variables.

#### Statistical analysis of CVL seeking-behavior

Our main hypothesis was that CVL diagnosis-seeking behavior would be associated with an occurrence of human and canine VL and proximity to the screening site. We defined the number of dogs tested on free demand per neighborhood as the outcome variable, with the predicted domestic canine population (see previous subsection) as an offset term. In addition, the urban neighborhoods of Rondonópolis were characterized according to the following explanatory variables: canine seropositivity for *Leishmania* spp. infection (i.e. the number of seropositive dogs for *Leishmania* spp. infection in both active and passive case detection divided by the total number of dogs screened for *Leishmania* spp. infection in both active and passive case detection), human VL incidence rate (cases/1,000 person-months) and tertiles of distance to the screening site (i.e. the linear distance—in meters—between the geographical coordinates of the centroids of each neighborhood to the USZ defined using QGIS 3.6.1 software [23]. A tertile distribution was chosen to capture the non-linear relationship between the outcome and the distance). The neighborhoods were also characterized according to the following potential demographic confounders: the natural logarithm of the mean monthly nominal income per PPH (in Brazilian minimum wages) and the natural logarithm of the number of inhabitants living in PPHs.

To minimize the risk of ecological fallacy (i.e. finding a correlation between two aggregated variables due to underlying confounding factors) and to capture a potential delay in the effect of the two time-dependent predictors (human VL incidence and canine seropositivity among screened dogs) on the outcome, we organized the data on the outcome, and the time-dependent predictors, in four different ways. First, as a baseline, all data per neighborhood were aggregated per year and time-dependent predictors were linked to the outcome in the same year. Second, all data per neighborhood were aggregated per month and time-dependent predictors were linked to the outcome in the same month. In both of these cases, there is a risk of ecological fallacy and no possibility of assessing a temporal association. Therefore, the third and the fourth approaches consider a delay between the time-dependent predictors and the outcome of 2 and 3 months, respectively. To be able to compare analyses based on these four variants of the dataset, we based all variants on exactly the same data, i.e. excluding data on the outcome from the first three months of the time series, as these could not be included in variant four (and partly in variant three).

A time delay of 2–3 months was chosen because the time between the onset of symptoms and the diagnosis of human VL in the municipality, as well as the time between the screening and euthanasia of a positive dog, was estimated in 25 and 41 days, respectively [24]. The offset term (the natural logarithm of the estimated dog population size), distance to the screening site, and demographic confounders were assumed to be constant over time and therefore were the same in all four variants.

We modelled the number of dogs tested on free demand per neighborhood using negative binomial regression. To account for within-neighborhood correlation in the dependent variable (*Y*) over time(*t*), we included a random intercept (*α*) for each neighborhood (*i*) [25].

$$\text{log}\left(Y_{it}\right)=\beta_{0}+\beta_{1}X_{1,it}+\ldots +\beta_{k}X_{k,it}+\;\text{log}\left(P_{i}\right)+\;\alpha_{i}$$ (1)

Here, *β*_0_ is the population-level intercept, *β*_1_,…,*β*_*k*_ are the regression coefficients for the *k* explanatory variables *X*_1_,…,*X*_*k*_, and *P* is the offset term (i.e. the number of dog-months at risk in the neighborhood).

To avoid biased fixed effects estimates due to correlation between unobserved factors (captured by the random intercepts) and observed predictors, we modelled fixed effects for explanatory variables that varied over time using the within-between approach [26–28]. In short, this means that time-varying neighborhood-level predictors were decomposed into two components: (1) a mean component (i.e., the mean over time for a neighborhood) and (2) a component representing the deviation from the neighborhood-level mean over time. Each component was then used as a predictor in the statistical model. The coefficient for a mean component represents the association between the outcome and differences in the predictor between neighborhoods, while the second coefficient represents the association between the outcome and differences in the predictor within neighborhoods over time. A variable was considered significantly associated with CVL diagnosis-seeking behavior if presented a *p-*value < 0.05. Regression analyses were performed on all four variants of the dataset.

All statistical analyses were executed in R 3.4.0 and R Studio 3.6.2 software [29] using the *mass* [30] and *lme4* [31] packages. Maps were produced using QGIS 3.6.1 software [23].

### Characterization of dog owners who sought screening for *Leishmania* spp. infection

To characterize the owners and their dogs enrolled at passive case detection, a descriptive survey was conducted based on a convenience sample of dog owners who spontaneously brought their animals to the USZ for serological screening for *Leishmania* spp. infection between October 2016 and September 2017. Individuals residing in Rondonópolis, older than 18 years, and who agreed to participate were included. After obtaining written consent, the individuals were interviewed based on a semi-structured questionnaire about socioeconomic aspects of the owner, characteristics of the animal and knowledge and attitudes on CVL (S1 Text).

Data entry was performed in duplicate and checked in Microsoft Office Excel 2013 (Microsoft Corp., Santa Rosa, CA, USA). A descriptive analysis was conducted with determination of absolute and relative frequencies for each variable.

## Results

**Fig 2:**
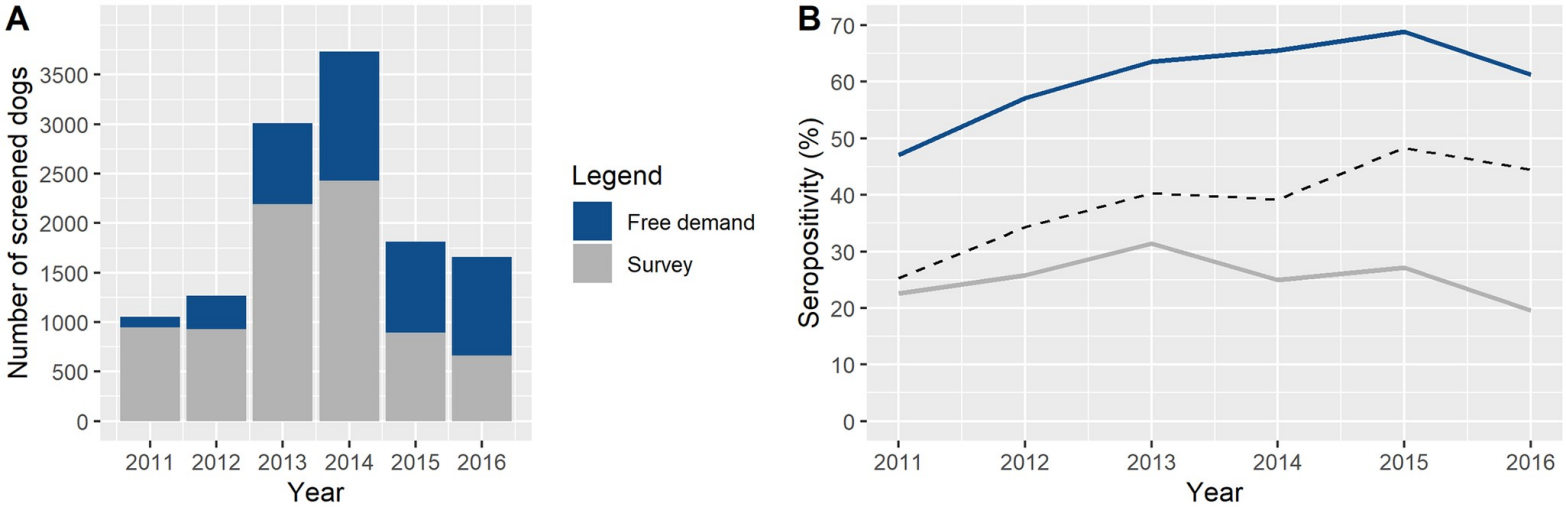
Serological screening for *Leishmania* spp. infection in the scope of public health in the municipality of Rondonópolis, state of Mato Grosso, Brazil, 2011–2016. (A) Stacked bar chart showing the annual absolute number of dogs screened on both free demand and surveys. (B) Line chart (not stacked) showing the annual seropositivity among the dogs screened on free demand and surveys. Dotted black line represents the overall seropositivity (free demand + surveys).

From 2011 to 2016, a total of 12,536 domestic dogs were submitted to serological screening for *Leishmania* spp. infection in the scope of public health in Rondonópolis. Of these, 64.2% (n = 8,048) were tested during serosurveys and 35.8% (n = 4,488) were screened on free demand. The annual number of dogs spontaneously tested increased considerably until a peak in 2014 (n = 1,416) followed by oscillating behavior in the last biennium. Active case detection also increased through to 2014 (n = 2,427), but steadily decreased afterwards. From 2015, the number of dogs screened by means of serosurveys became lower than on free demand (Fig 2A).

Among the tested animals, 12,138 presented a conclusive serology and 4,857 were found to be positive for *Leishmania* spp. infection, resulting in an overall seropositivity of 40.0% (95%-confidence interval (CI) = 39.1%– 40.9%). Canine seropositivity was higher among dogs tested on free demand (63.9%; CI = 62.5%– 65.3%) compared to those screened during serosurveys (26.4%; CI = 25.4%– 27.3%). This large difference was observed over all the evaluated years; while the annual seropositivity among passive case detection ranged from 47.1% to 68.9%, the values related to active case detection ranged from 19.6% to 31.4% (Fig 2B).

**Fig 3:**
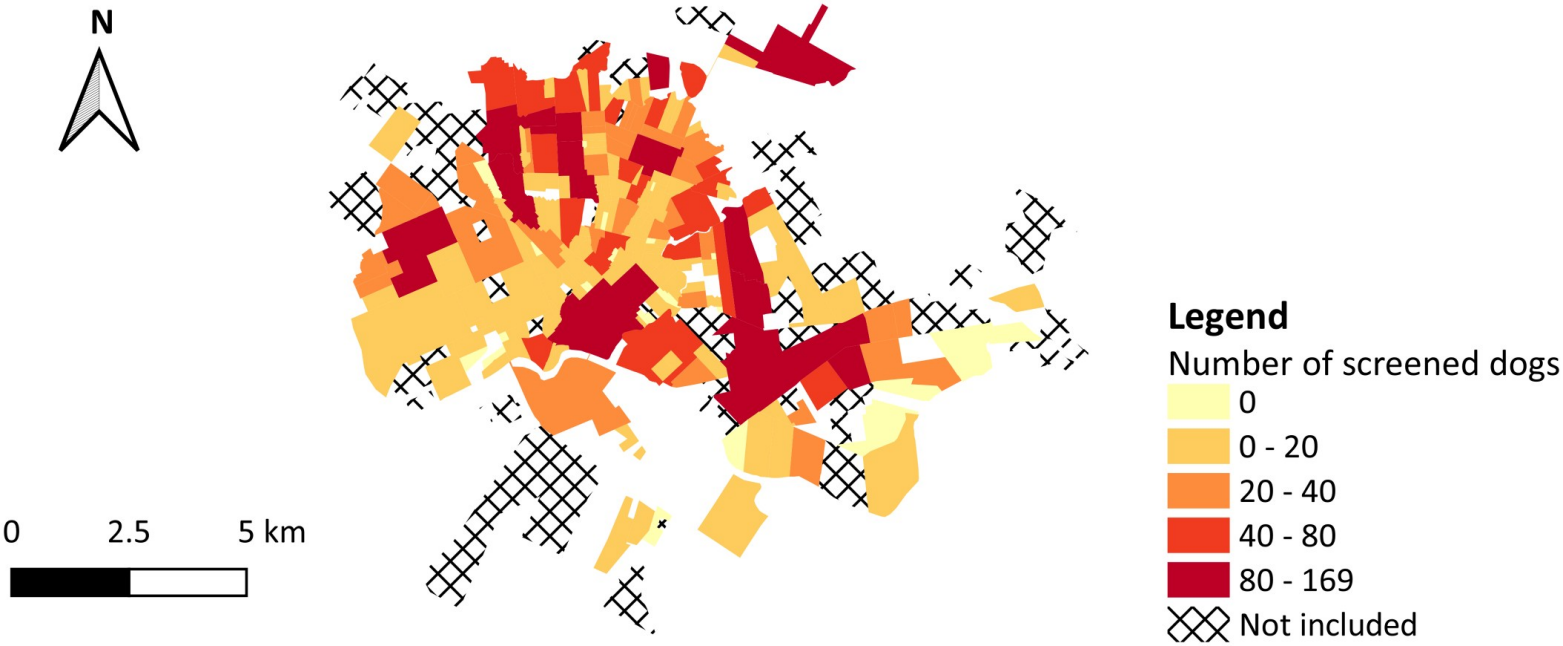
Absolute number of dogs screened for canine visceral leishmaniasis on free demand per urban neighborhood in the municipality of Rondonópolis, state of Mato Grosso, Brazil, 2011–2016. In the legend, colors represent ranges of neighborhood-level values, where the upper bounds are included within interval. Digital georeferenced database of the neighborhoods was provided by the Municipal Health Department of Rondonópolis.

In 160 of 183 evaluated neighborhoods of Rondonópolis, at least one dog owner spontaneously presented their dog for screening for *Leishmania* spp. infection during the study period. The cumulative number of dogs tested on free demand per neighborhood ranged from zero to 169 animals; *Parque Universitário* (n = 169), *Vila Operária* (n = 148), and *Jardim Liberdade* (n = 137) led the records (Fig 3).

**Fig 4:**
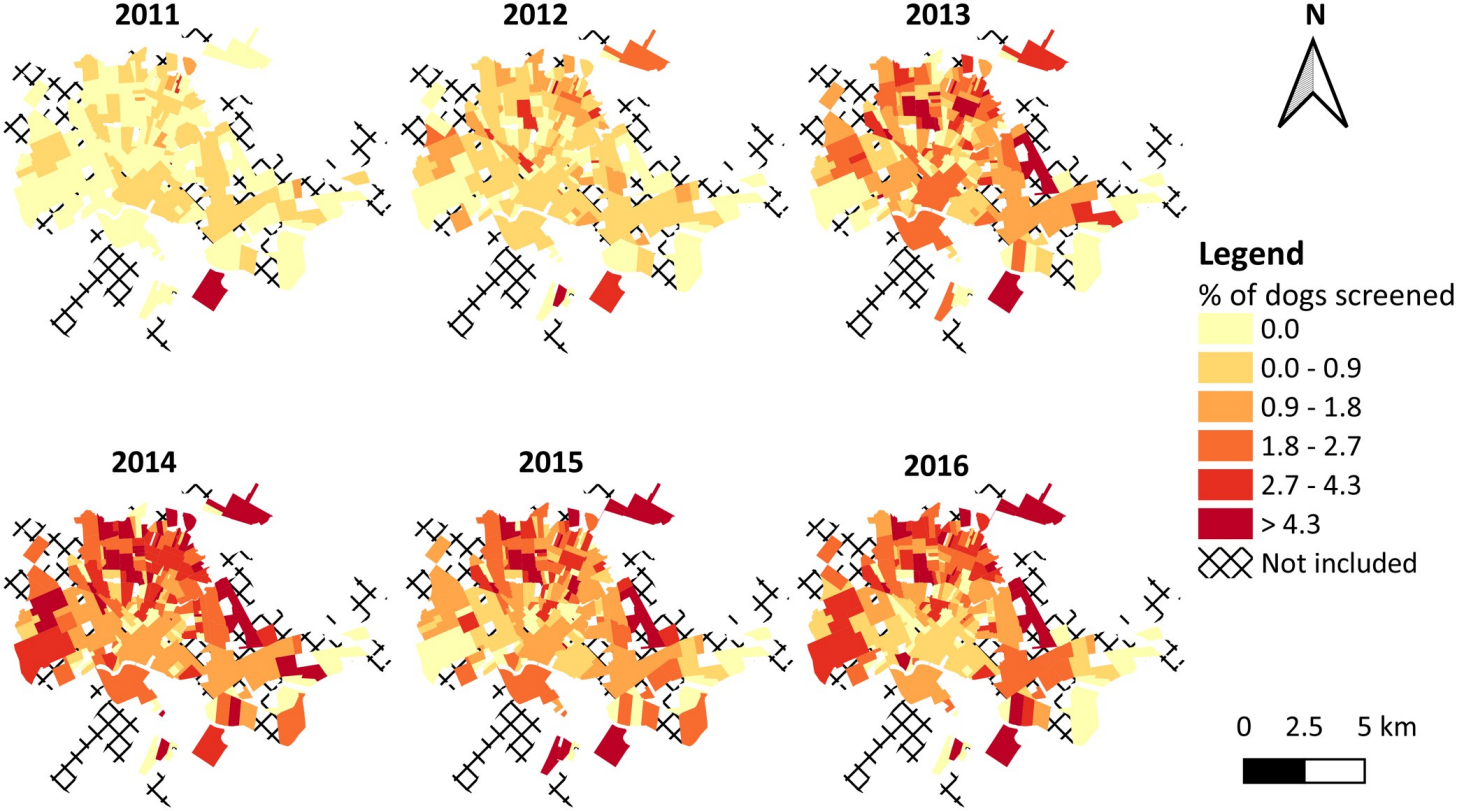
Annual percentage of dogs screened for *Leishmania* spp. infection among the estimated canine population size in urban neighborhoods of the municipality of Rondonópolis, state of Mato Grosso, Brazil, 2011–2016. In the legend, colors represent ranges of neighborhood-level values, where the upper bounds are included within interval. Digital georeferenced database of the neighborhoods was provided by the Municipal Health Department of Rondonópolis.

The total number of domestic dogs for Rondonópolis was estimated at 56,366 animals according to demographic predictors (S1 Table) using the statistical model for the association between demographic predictors and number of dogs per household (S2 Table). The canine population size per neighborhood ranged from 10 to 2094 individuals (S1 Fig). Fig 4 shows the annual percentage of dogs screened on free demand per neighborhood, which widely varied (range 0.0 to 36.0%) and increased over the years in general. Highest percentage values were found among the neighborhoods located in the northern and western regions of the municipality.

**Fig 5:**
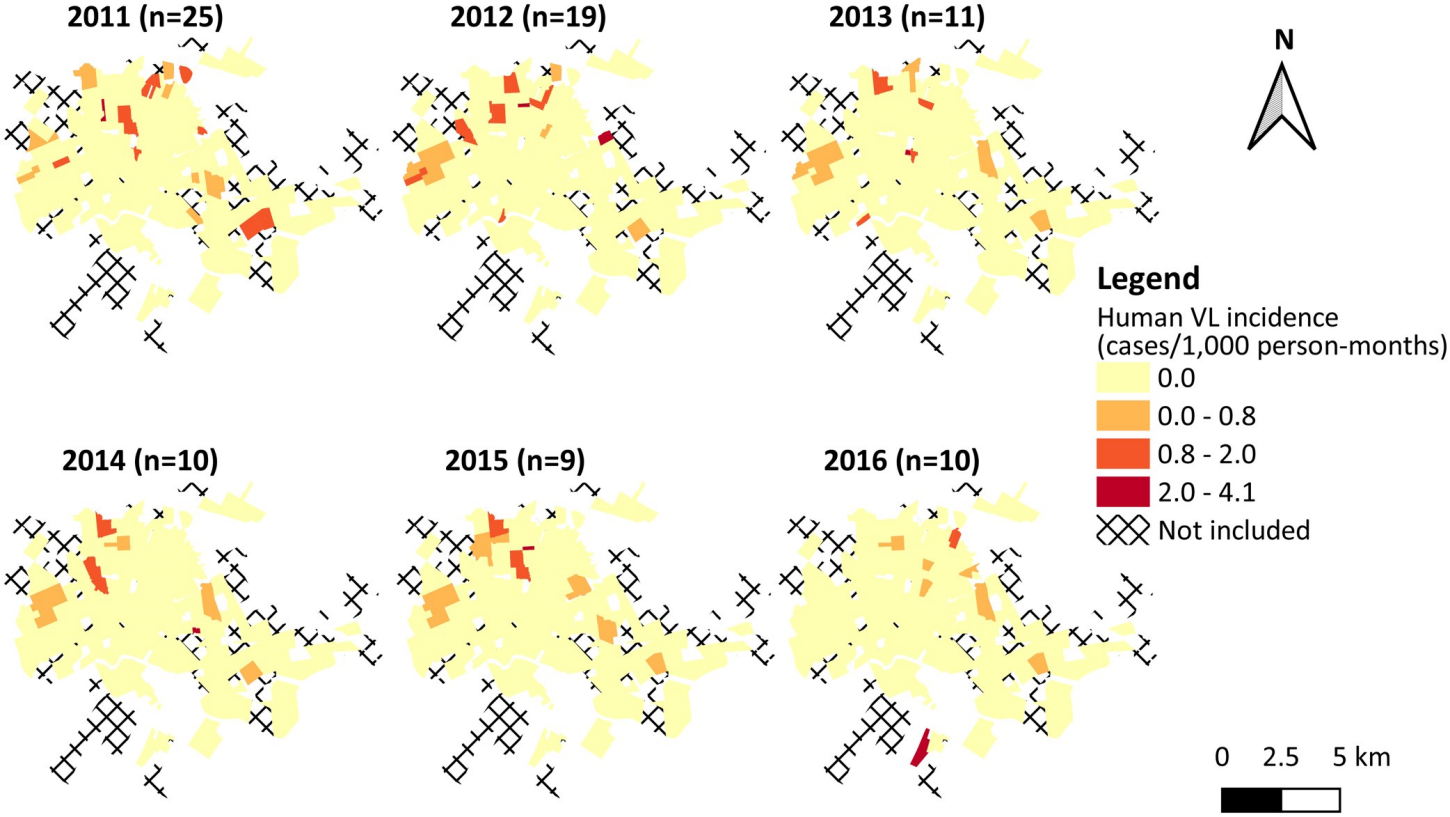
Human visceral leishmaniasis (VL) in urban neighborhoods of the municipality of Rondonópolis, state of Mato Grosso, Brazil, 2011–2016. Human VL incidence and the number of cases reported in each year. In the legend, colors represent ranges of neighborhood-level values, where the upper bounds are included within interval. Digital georeferenced database of the neighborhoods was provided by the Municipal Health Department of Rondonópolis.

Regarding human VL, 47 neighborhoods of the municipality reported a total of 84 cases, which constituted a total incidence of 0.46 cases/1,000 person-months over all neighborhoods. The annual human VL incidence rates per neighborhood ranged from 0.0 to 4.1 cases/1,000 person-months (mean 0.07 cases/1,000 person-months with standard deviation (SD) 0.32 cases/1,000 person-months). Between 2011 and 2012, the number of cases as well as the number of neighborhoods with at least one VL case were higher compared to the following years (Fig 5).

**Fig 6:**
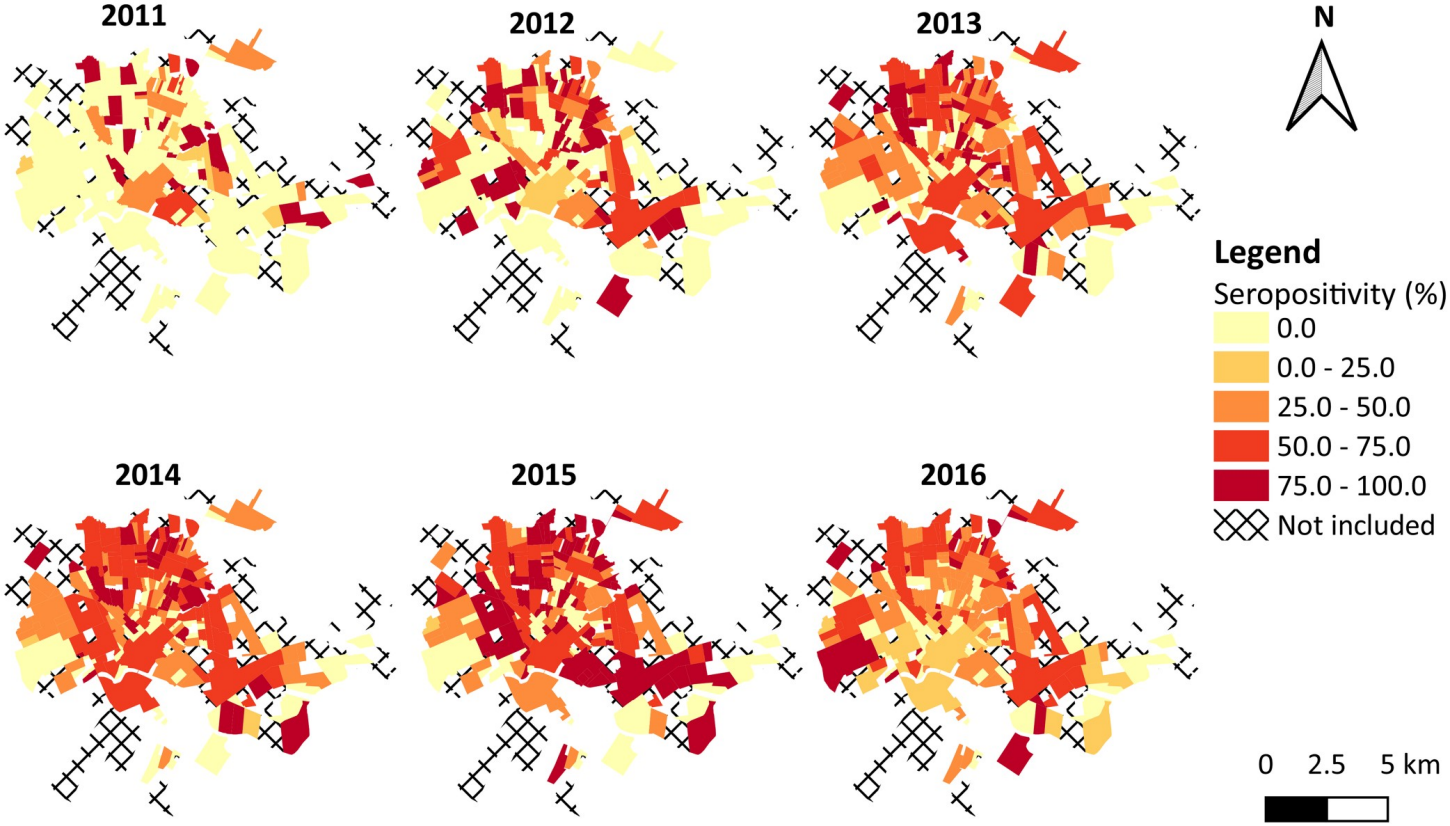
*Leishmania* spp. infection among dogs in urban neighborhoods of the municipality of Rondonópolis, state of Mato Grosso, Brazil, 2011–2016. Seropositivity for *Leishmania* spp. infection in both active and passive case detection. In the legend, colors represent ranges of neighborhood-level values, where the upper bounds are included within interval. Digital georeferenced database of the neighborhoods was provided by the Municipal Health Department of Rondonópolis.

The mean (SD) annual canine seropositivity per neighborhood in both active and passive case detection was 36.2% (37.5%), and the number of areas with high seropositivity increased over the years, especially after 2013 (Fig 6). The linear distance between the geographical coordinates of the centroids of each neighborhood and the screening site ranged from 308.2 to 10,874.0 m, with mean (SD) of 5,309.2 (2,146.2) m (Fig 1B).

Table 1 presents the estimated model coefficients for factors associated with CVL diagnosis-seeking behavior in Rondonópolis in the four variants of the dataset. An increase in the canine seropositivity for *Leishmania* spp. infection within neighborhoods as well as differences in canine seropositivity between neighborhoods were strongly associated with an increase in CVL diagnosis seeking-behavior. The magnitude of the coefficients within neighborhoods decreased in the models with time delay between this predictor and the outcome, but remained significant. On the other hand, an increase in human VL incidence within neighborhoods was associated with a decrease in CVL diagnosis-seeking behavior in the same year. However, this effect was not significant in models considering the data on a monthly basis or when considering a time delay between predictor and outcome. There was no significant effect of variation in VL incidence between neighborhoods on CVL diagnosis-seeking behavior. We found that the number of dogs screened on free demand was significantly higher among those neighborhoods located closer to the screening site (≤ 4,410 m, i.e. the 1^st^ tertile of distance) when compared to those more distant (> 4,410 m away, i.e. the 2^nd^ and 3^rd^ tertiles of distance; there was no significant difference between the 2^nd^ and the 3^rd^ tertiles). Further, the mean income of the neighborhood was positively associated with the CVL diagnosis-seeking behavior, but only significantly in the model with monthly analysis without a time delay. The impact of income was larger for neighborhoods with relatively low income (i.e. coefficients between zero and one for the natural logarithm of mean income).

**Table 1:** Mixed effects regression models for the number of dogs screened on free demand for *Leishmania* spp. infection (canine visceral leishmaniasis diagnosis-seeking behavior) in the scope of public health in the neighborhoods of Rondonópolis, Mato Grosso, Brazil (2011–2016).

Variable	Dataset arrangement											
	Same year^a^			Same month^b^			Two-month delay^c^			Three-month delay^d^		
	β	SE	*p*-value	β	SE	*p*-value	β	SE	*p*-value	β	SE	*p*-value
Canine seropositivity for *Leishmania* spp. infection (%)												
*Δ within-neighborhoods*	1.600	0.122	<0.001*	2.357	0.045	<0.001*	0.409	0.055	<0.001*	0.350	0.055	<0.001*
*Δ between-neighborhoods*	2.991	0.398	<0.001*	6.246	0.497	<0.001*	6.394	0.544	<0.001*	6.434	0.547	<0.001*
Human VL incidence (cases / 1,000 person-months)												
*Δ within-neighborhoods*	-0.433	0.134	0.001*	-0.147	0.239	0.538	-0.463	0.273	0.089	-0.053	0.236	0.824
*Δ between-neighborhoods*	0.719	0.421	0.088	2.111	3.426	0.538	3.659	3.602	0.310	3.932	3.845	0.307
Distance to the screening site (1^st^ versus 2^nd^ + 3^rd^ tertiles)^e^^,^^f^	-0.319	0.146	0.029*	-0.220	0.096	0.022*	-0.320	0.101	0.001*	-0.328	0.101	0.001*
Logarithm of mean monthly nominal income per PPH (in terms of Brazilian minimum wages)^f^^,^^g^	0.140	0.156	0.371	0.216	0.106	0.042*	0.195	0.111	0.078	0.196	0.111	0.078
Logarithm of human population size ^f^	-0.325	0.068	<0.001*	-0.711	0.057	<0.001*	-0.649	0.060	<0.001*	-0.646	0.060	<0.001*
Intercept	-5.218	0.264	<0.001*	-6.989	0.163	<0.001*	-6.685	0.170	<0.001*	-6.673	0.170	<0.001*
Number of observations	1098			12627			12627			12627		
Number of neighborhoods	183			183			183			183		

In addition to the reported predictors, the model contained an offset for the natural logarithm of the number of dog-years at risk.

SE: standard error. PPH: private permanent household.

^a^ Data on outcome and predictors aggregated by year.

^b^ Data on outcome and predictors aggregated by month, without a time delay between outcome and predictors.

^c^ Data on outcome and predictors aggregated by month, with a two-month delay between outcome and predictors.

^d^ Data on outcome and predictors aggregated by month, with a three-month delay between outcome and predictors.

^e^ Refers to the linear distance (in meters) between the centroids of each neighborhood to the local Unit of Surveillance in Zoonosis: 1^st^ tertile (< 4,410 m) / 2^nd^ tertile (4,411–6,262 m) / 3^rd^ (6,263–10,874 m).

^f^ Variables considered to be constant over time.

^g^ Brazilian minimum wage (2010) = US$ 116.2 (R$ 510).

Of the 804 animals that were screened for *Leishmania* spp. infection on free demand at the USZ from October 2016 to September 2017, 93 dog owners who altogether owned 99 animals were interviewed. Individuals with higher family income (> one Brazilian minimum wage) and high levels of schooling prevailed among the owners enrolled on free demand. Their mean (interquartile range—IQR) age was 44.5 (20) years. Further, most of them were aware about basic aspects of VL, such as its way of transmission and prevention. Regarding VL prevention, the majority of dog owners named at least one measure, and environmental management was cited with the highest frequency. On the other hand, the recommendation of canine euthanasia in controlling VL was unknown by almost half of the participants (Table 2).

**Table 2:** Frequency distribution of dog owners who attended the screening site seeking for canine visceral leishmaniasis (CVL) diagnosis according to sociodemographic characteristics and knowledge on VL.

Variable	n	%
**Sex**		
Female	50	53.8
Male	43	46.2
**Age group (years)** ^a^		
18–40	36	38.7
40–60	42	45.2
≥ 60	12	12.9
Not reported	3	3.2
**Family income (Brazilian minimum wages)** ^a^ ^,^ ^b^		
≤ 1	10	10.8
1–3	36	38.7
> 3	43	46.2
Not reported	4	4.3
**Educational level**		
Illiterate or primary education	11	11.8
Elementary education	23	24.7
High school	46	49.5
College	10	10.8
Not reported	3	3.2
**How VL’s agent is transmitted?**		
Insect bite	80	86.0
Do not know	13	14.0
**How to prevent VL?** ^c^		
Environmental management	46	49.5
Avoiding standing water	17	18.3
Insecticide spraying	15	16.1
Dog collar or topical repellent	15	16.1
Vaccination	3	3.2
Others	6	6.4
Do not know	21	22.6
**Why canine euthanasia of infected dogs is recommended?**		
To avoid the transmission of VL’s agent to humans and / or animals	29	31.2
Because CVL has no cure	10	10.7
To avoid the transmission of VL’s agent to humans and / or animals and because CVL has no cure	8	8.6
Others	4	4.3
Do not know	42	45.2

Rondonópolis, Mato Grosso State, Brazil (2016–2017).

^a^The upper bounds are included in each class interval.

^b^Brazilian minimum wage (2016) = US$ 276.7 (R$ 880).

^c^For this variable, the number of responses per participant was unlimited.

Among the 99 animals screened on free demand, the mean (IQR) age was 38.4 (30) months and the majority were companion dogs. In addition, most of the animals (73.7%) presented at least one apparent clinical manifestation suggestive of CVL that lasted for more than 30 days (mean (SD): 131.1 (179.0) days). Because of these clinical signs, half of the dogs had already undergone treatment. Thus, clinical signs consistent with CVL were the most frequent motivation for a dog owner to seek diagnosis for the disease (68.7%); other common reasons were routine/prevention procedure (15.1%) and existence of dogs with CVL in the neighborhood (9.1%). Most owners indicated they would opt for euthanasia in case of positivity for *Leishmania* spp. infection (Table 3).

**Table 3:** Frequency distribution of dogs who were screened for *Leishmania* spp. infection on free demand in the scope of public health according to physical characteristics and attitudes of their respective owners.

Variable	n	%
**Gender**		
Female	53	53.5
Male	46	46.5
**Finality**		
Companion	86	86.9
Guard dog	13	13.1
**Age group (months)** ^a^		
< 12	14	14.2
12–24	22	22.2
24–36	19	19.2
≥ 36	42	42.4
Not reported	2	2.0
**Apparent signs consistent with canine visceral leishmaniasis (CVL)**		
Present	73	73.7
Absent	26	26.3
**Time presenting apparent signs consistent with CVL (days)** ^a^		
< 30	12	12.1
30–90	21	21.2
90–180	12	12.1
≥ 180	19	19.2
Not applicable / not reported	35	35.4
**Previously treated for the signs consistent with CVL**		
Yes	50	50.5
No	23	23.2
Not applicable	26	26.3
**Reason for performing serological screening on free demand**		
Presence of suggestive CVL signs	68	68.7
Routine / prevention	15	15.1
Existence of dogs with CVL in the neighborhood	9	9.1
Referral from someone else	6	6.1
Existence of humans with VL in the neighborhood	1	1.0
**Attitude in case of seropositivity**		
Canine euthanasia	58	58.6
Dog treatment	23	23.2
Use of insecticide-impregnated dog collars	3	3.0
Do not know	15	15.2

Rondonópolis, Mato Grosso State, Brazil (2016–2017).

^a^The lower bounds are included in each class interval.

## Discussion

This is the first study addressing CVL passive case detection on request of individual dog owners in the scope of Brazilian public health. In addition to a multilevel approach considering urban neighborhoods of a relevant endemic area [16,32] as analytical units, we conducted a descriptive survey with dog owners to support the interpretation of ecological results. In summary, the number of dogs screened for *Leishmania* spp. infection on free demand increased over years with high seropositivity in the municipality of Rondonópolis. At the neighborhood-level, CVL diagnosis-seeking behavior was associated with the occurrence of *Leishmania* spp. canine infection and proximity to the screening site. At the individual level, dog owners with better socioeconomic conditions and dogs with apparent clinical manifestations prevailed among those individuals who sought for screening. Given the lack of a neighborhood-level association between human VL incidence and diagnosis-seeking behavior, as well as the facts that (1) almost half of the dog owners were not aware of the reason for canine euthanasia in case of CVL, and (2) most owners presented their dogs after an extended period of symptoms, it seems that awareness about the link between VL and CVL was limited.

The number of dogs tested on free demand was strongly positively associated with canine seropositivity for *Leishmania* spp. infection in all evaluated variants of the dataset. This relationship suggests some degree of causality, since a significant association of the outcome with within-neighborhood variation was observed in both models accounting for a time delay. This association may be explained by the consolidated circulation of *Leishmania* spp. infection among dogs observed across the neighborhoods of Rondonópolis [33]. It is likely that the widespread distribution of infected dogs, especially those with apparent signs of CVL, motivates owners to seek care for their animals. Indeed, a previous occurrence of CVL cases in the neighborhood was already demonstrated as associated with improved knowledge, attitudes and practices regarding the disease among dog owners from Rondonópolis [34]. In the present study, a portion of interviewed dog owners reported seeking for screening as a routine/prevention procedure or due to the presence of canine cases in their neighborhood.

Our analyses show no consistent association between human VL incidence and CVL diagnosis-seeking behavior of dog owners at the neighborhood level. This association was only statistically significant when considering the data aggregated to the year and without time delay. As the latter variant of our analysis is more prone to ecological fallacy, and its results were not reproduced by analyzing the data at a more detailed time scale, we conclude that this association is likely spurious, either as a chance finding or through confounding due to aggregation (ecological fallacy).

Although CVL passive case detection can be a way for performing serological screening in areas with scarce resources, the application of such results for guiding public health measures must be done with caution. As we noticed, seropositivity for *Leishmania* spp. infection was clearly higher among dogs tested on free demand, compared to animals screened by surveys. Corroborating this observation, a population-based serosurvey recently detected an overall CVL seroprevalence of 19.2% in Rondonópolis [17]. This value was substantially lower than any annual seropositivity observed here in dogs screened under free demand (> 47%).

The high seropositivity for *Leishmania* spp. infection within the scope of passive case detection, along with the well-known association between CVL clinical manifestations and reactivity of serological tests [35–37], suggest that CVL diagnosis-seeking behavior is related to animals presenting advanced clinical stages of the disease. This assumption was corroborated by the descriptive survey, since most of the animals presented at least one clinical manifestation of CVL, which was pointed out by dog owners as the main motivation for seeking diagnosis. In this context, despite its dependence on the inclination of dog owners to seek diagnosis for their dogs, public health surveillance units should consider CVL diagnosis on free demand an important and useful indicator of the occurrence of CVL, although it has three main challenges which we will discuss in more detail next.

The present study demonstrated that the geographic location of the screening site might represent an obstacle to CVL diagnosis seeking-behavior. A decrease in the number of dogs screened was associated with an increasing distance from neighborhoods to the USZ in all analyzed variants of the dataset. In Rondonópolis, the USZ is located away from the urban center with limited public transportation coverage. Previous investigations have already reported the distance and the difficulty in accessing the screening sites as factors that contribute to late diagnosis [38–39] and treatment [40] of other diseases. As an attempt to enhance CVL diagnosis-seeking behavior, multiple screening sites options should be made available in the area, especially in more central and/or easily accessible zones. For these purposes, we suggest using existing primary healthcare facilities that are supported by a multidisciplinary team of the Family Health Support Centers (*Núcleos de Apoio à Saúde da Família*). These sometimes include veterinarians who could facilitate canine blood collection for passive case detection [41]. A potential challenge here might be that it would take more effort to ensure completeness and quality of data for monitoring purposes, as data collection would no longer be centralized in the USZ. In an optimistic scenario, even the point-of-care test recommended by the VLSCP for screening (TR DPP) could be performed in these sites. We also recommend local government support for free CVL testing through local private clinics (with clear rules on how and when dogs are eligible for testing).

The second challenge is that screening for *Leishmania* spp. infection on free demand is under-used by socio-economically challenged groups, who are at higher risk of VL [42]. In Rondonópolis, it was recently observed that the occurrence of canine infection by *Leishmania* spp. is associated with lower socio-economic position of dog owners [43]. In contrast, most of the individuals interviewed in the present study while attending the screening site seeking for CVL diagnosis presented a better family income and a high level of education. In our neighborhood-level analysis, we did not find a statistically significant association between CVL diagnosis-seeking behavior and mean monthly nominal income per PPH, although the direction of the association did fit the notion of low uptake by socio-economically challenged groups. It is possible that a large portion of infected dogs from the municipality, which are those animals living in the poorest neighborhoods and owned by the poorest individuals, are not covered by passive case detection. Therefore, we strongly recommend that the aforementioned implementation of multiple screening sites must prioritize neighborhoods with the worst socioeconomic indicators in order to enhance the coverage of screening among the poorest populations.

The third main challenge for passive case detection is the seemingly low awareness about the link between CVL and VL detected among dog owners. This was previously observed in the area among primary health care professionals [44]. Coura-Vital et al. [45] reported elsewhere in Brazil that only one third of dog owners knew the role of dogs in the transmission of VL’s agent to humans. More recently, a household-based survey observed poor knowledge, attitudes and practices regarding CVL among dog owners from Rondonópolis [34]. It is likely that these gaps negatively impact the seeking of CVL diagnosis at screening sites. In our study, only one dog owner sought screening for *Leishmania* spp. infection because of a previous human VL cases in the neighborhood. Also, alarmingly, most of the affected dogs enrolled in our descriptive survey presented such clinical manifestations for long periods. Consequently, infected dogs may contribute to the maintenance of local transmission cycles of *L*. *infantum* involving other animals, sand flies and humans nearby [46]. Thus, health educational programs aimed at population awareness about the importance of routine serological screening for *Leishmania* spp. infection in endemic areas should be prioritized. It is important that dog owners are aware of the infection status of their animals, regardless of the occurrence of clinical manifestations. For that, we suggest the continuous training of community health workers and school teachers, who can act as spreaders of knowledge within their communities. Also, the integration of health education activities aimed at multiple endemic zoonoses must be encouraged, such as rabies and VL [34].

As this study was based on a secondary analysis of routinely collected data, we faced two important technical challenges. First of all, there was no data available on the size of the domestic canine population in Rondonópolis, for which our estimates may have deviated somewhat from reality. However, we consider our estimates robust enough, given that variation between neighborhoods in terms of the estimated total number of dogs was mostly driven by actual data on the number of households in each neighborhood (S1 Table). Second, the use of neighborhood-level data (at which level data on relevant predictors were available) introduces the risk of ecological fallacy. Also known as Simpson’s paradox, this may cause associations to be found that do not hold at lower levels of aggregation. To reduce this risk, we (1) explicitly modelled trends over time within neighborhoods by means of mixed effects regression, (2) checked that our findings were robust to various time delays for the association between predictors and outcome, and (3) supplemented our neighborhood-level analysis with an analysis of subsample of individual-level questionnaire data. As for this subsample, which was based on a convenience sample of dog owners, it is possible that higher socio-economic groups were overrepresented in the sample. This may have inflated our estimates of self-reported knowledge about the public health relevance of canine VL, further highlighting the need for policy action. A last point of note is that we selected variables based on forward selection, a method that is known to inflate Type I errors [47]. However, this is not necessarily problematic given the exploratory nature of our research and the fact that our findings seem to follow common sense and fit well with existing literature.

In conclusion, passive case detection is an important tool to diagnose CVL and monitor its occurrence in the population. However, its implementation and uptake are most likely challenged by distance to the screening site, low socioeconomic status and low population awareness about CVL and its epidemiological relevance. Therefore, public health authorities and decision-makers should consider the implementation of multiple accessible screening sites, prioritization of socio-economically disadvantaged areas and the implementation of health educational activities for the community. In parallel, CVL active case detection must be conducted as a surveillance tool to guide control actions. Taken together, these measures can enhance the screening of canine reservoirs and, hopefully, improve the control of VL.

## Supporting information

S1 Text Questionnaire about socioeconomic aspects of the dog owner, characteristics of the animal and knowledge and attitudes on canine visceral leishmaniasis. This version has been adapted and translated from Portuguese.
(DOCX)

S1 Table Predictor values and the predicted domestic canine population in urban neighborhoods of the municipality of Rondonópolis, state of Mato Grosso, Brazil. Data on predictors were extracted from the last Demographic Census [14].
(XLSX)

S2 Table Negative binomial regression coefficients for the number of domestic dogs per household in each primary sampling unit as a function of demographic predictors. Data were extracted from the National Health Survey [19] for the state of Mato Grosso, Brazil.
(DOCX)

S1 Fig Predicted domestic canine population in urban neighborhoods of the municipality of Rondonópolis, state of Mato Grosso, Brazil. In the legend, colors represent ranges of neighborhood-level values, where the upper bounds are included within interval. Digital georeferenced database of the neighborhoods was provided by the Municipal Health Department of Rondonópolis.
(TIF)

## References

[1] Ready PD. Epidemiology of visceral leishmaniasis. Clin Epidemiol. 2014;6:147–154. doi: 10.2147/clep.s44267 24833919 PMC4014360

[2] Alvar J, Vélez ID, Bern C, Herrero M, Desjeux P, Cano J, et al. Leishmaniasis worldwide and global estimates of its incidence. PLoS One. 2012;7(5):e35671. doi: 10.1371/journal.pone.0035671 22693548 PMC3365071

[3] Reis LLD, Balieiro AADS, Fonseca FR, Gonçalves MJF. Changes in the epidemiology of visceral leishmaniasis in Brazil from 2001 to 2014. Rev Soc Bras Med Trop. 2017;50(5):638–645. doi: 10.1590/0037-8682-0243-2017 29160510

[4] Ursine RL, Dias JVL, Morais HA, Pires HHR. Human and canine visceral leishmaniasis in an emerging focus in Araçuaí, Minas Gerais: spatial distribution and socio-environmental factors. Mem Inst Oswaldo Cruz. 2016;111(8):505–511. doi: 10.1590/0074-02760160133 27384080 PMC4981116

[5] Arruda RMF, Cardoso DT, Teixeira-Neto RG, Barbosa DS, Ferraz RK, Morais MHF, et al. Space-time analysis of the incidence of human visceral leishmaniasis (VL) and prevalence of canine VL in a municipality of southeastern Brazil: Identification of priority areas for surveillance and control. Acta Trop. 2019;197:105052. doi: 10.1016/j.actatropica.2019.105052 31233726

[6] Peixoto HM, de Oliveira MRF, Romero GAS. Serological diagnosis of canine visceral leishmaniasis in Brazil: systematic review and meta-analysis. Trop Med Int Health. 2015;20(3):334–352. doi: 10.1111/tmi.12429 25403359

[7] Brasil. Ministério da Saúde. Secretaria de Vigilância em Saúde. Departamento de Vigilância Epidemiológica. Manual de Vigilância e Controle da Leishmaniose Visceral; 2006. https://bvsms.saude.gov.br/bvs/publicacoes/manual_vigilancia_controle_leishmaniose_visceral.pdf.

[8] Góes MAO, Melo CM, Jeraldo VLS. Time series of visceral leishmaniasis in Aracaju, State of Sergipe, Brazil (1999 to 2008): human and canine aspects. Rev Bras Epidemiol. 2012;15(2):298–307. doi: 10.1590/s1415-790x2012000200007 22782095

[9] Barbosa DS, Belo VS, Rangel ME, Werneck GL. Spatial analysis for identification of priority areas for surveillance and control in a visceral leishmaniasis endemic area in Brazil. Acta Trop. 2014;131:56–62. doi: 10.1016/j.actatropica.2013.12.002 24342506

[10] Brito VN, Oliveira CM, Lazari P, Sousa VRF. Epidemiological aspects of visceral leishmaniasis in Jaciara, Mato Grosso, Brazil, 2003 to 2012. Rev Bras Parasitol Vet. 2014 Mar;23(1):63–68. doi: 10.1590/s1984-29612014008 24728362

[11] Morais MHF. Avaliação das atividades de controle da leishmaniose visceral em Belo Horizonte, Minas Gerais, 2006–2011. Epidemiol Serv Saude. 2015;24(3):485–496. doi: 10.5123/S1679-49742015000300014

[12] Escobar TA, Döwich G, Zuravski L, Cantele LC, Duarte CA, Lübeck I. Risk factors associated to canine visceral leishmaniasis in Uruguaiana city, Brazil. Semin Cienc Agrar. 2018;39(1):211–220. doi: 10.5433/1679-0359.2018v39n1p211

[13] Instituto Brasileiro de Geografia e Estatística (IBGE); 2021. https://cidades.ibge.gov.br/brasil/mt/rondonopolis.

[14] Instituto Brasileiro de Geografia e Estatística (IBGE). Censo Demográfico; 2010. https://sidra.ibge.gov.br/pesquisa/censo-demografico/demografico-2010/universo-caracteristicas-da-populacao-e-dos-domicilios.

[15] Carvalho AG, Kuhn AL, Dias JV, Santos ES, Luz JGG. Análise da ocorrência de leishmaniose visceral humana no estado brasileiro de Mato Grosso: Um panorama espacial e demográfico atualizado (2001–2016). In: Ribeiro EA, Beceyro C, Santos FO, editors. Abordagens Geográficas da Vigilância, Prevenção e Promoção da Saúde. Florianópolis: Editora IFC; 2019. pp.30–38.

[16] Luz JGG, Naves DB, Carvalho AG, Meira GA, Dias JVL, Fontes CJF. Visceral leishmaniasis in a Brazilian endemic area: an overview of occurrence, HIV coinfection and lethality. Rev Inst Med Trop Sao Paulo. 2018;60:e12. doi: 10.1590/S1678-9946201860012 29538509 PMC5962093

[17] Carvalho AG, Luz JGG, Rodrigues LD, Dias JVL, Fontes CJF. High seroprevalence and peripheral spatial distribution of visceral leishmaniasis among domestic dogs in an emerging urban focus in Central Brazil: a cross-sectional study. Pathog Glob Health. 2018;112(1):29–36. doi: 10.1080/20477724.2018.1438229 29460695 PMC6056830

[18] Brasil. Ministério da Agricultura, Pecuária e Abastecimento. Coordenação de Fiscalização de Produtos Veterinários. Nota Técnica n° 11/2016/CPV/DFIP/SDA/GM/MAPA. Autoriza o registro do produto Milteforan; 2016. http://www.sbmt.org.br/portal/wp-content/uploads/2016/09/nota-tecnica.pdf.

[19] Instituto Brasileiro de Geografia e Estatística (IBGE). Pesquisa Nacional de Saúde; 2013. http://www.ibge.gov.br/home/estatistica/populacao/pns/2013/.

[20] Brasil. Ministério da Saúde. Secretaria de Vigilância em Saúde. Departamento de Vigilância das Doenças Transmissíveis. Coordenação Geral de Doenças Transmissíveis. Coordenação Geral de Laboratórios de Saúde Pública. Nota Técnica Conjunta n° 1, de 2011. Esclarecimentos sobre substituição do protocolo diagnóstico da leishmaniose visceral canina (LVC); 2011. http://www.sgc.goias.gov.br/upload/arquivos/2012-05/nota-tecnica-no.-1-2011_cglab_cgdt1_lvc.pdf.

[21] Martins CM, Mohamed A, Guimarães AMS, Barros CC, Pampuch RS, Svoboda W, et al. Impact of demographic characteristics in pet ownership: modeling animal count according to owners income and age. Prev Vet Med. 2013;109(3–4):213–218. doi: 10.1016/j.prevetmed.2012.10.006 23154107

[22] Souza-Júnior PRB, Freitas MPS, Antonaci GA, Szwarcwald CL. Sampling Design for the National Health Survey, 2013. Epidemiol Serv Saúde. 2015; 24(2):207–216. doi: 10.5123/S1679-49742015000200003

[23] QGIS.org (2019). QGIS Geographic Information System. Open Source Geospatial Foundation Project. http://qgis.org.

[24] Luz JGG, Carvalho AG, Naves DB, Dias JVL, Fontes CJF. Where, when, and how the diagnosis of human visceral leishmaniasis is defined: answers from the Brazilian control program. Mem Inst Oswaldo Cruz. 2019;114:e190253. doi: 10.1590/0074-02760190253 31664313 PMC6821129

[25] Austin PC, Stryhn H, Leckie G, Merlo J. Measures of clustering and heterogeneity in multilevel Poisson regression analyses of rates/count data. Stat Med. 2018;37(4):572–589. doi: 10.1002/sim.7532 29114926 PMC5813204

[26] Dieleman JL, Templin T. Random-effects, fixed-effects and the within-between specification for clustered data in observational health studies: a simulation study. PLoS One. 2014;9(10):e110257. doi: 10.1371/journal.pone.0110257 25343620 PMC4208783

[27] Dieleman JL, Templin T. Correction: Random-Effects, Fixed-Effects and the within-between Specification for Clustered Data in Observational Health Studies: A Simulation Study. PLoS One. 2016;11(5):e0156508. doi: 10.1371/journal.pone.0156508 27218254 PMC4878732

[28] Bell A, Jones K. Explaining fixed effects: Random effects modeling of time-series cross-sectional and panel data. Polit Sci Res Methods. 2014;3(1):133–153. doi: 10.1017/psrm.2014.7

[29] R Studio Team (2015). RStudio: Integrated Development for R. RStudio, Inc., Boston, MA URL. http://www.rstudio.com/

[30] Venables WN, Ripley BD. Modern Applied Statistics with S. 4th ed. New York: Springer; 2002.

[31] Bates D, Mächler M, Bolker B, Walker S. Fitting linear mixed-effects models using lme4. J Stat Softw. 2015;67(1):1–48. doi: 10.18637/jss.v067.i01

[32] Luz JGG, Carvalho AG, Naves DB, Dias JVL, Fontes CJF. Are backyard characteristics relevant factors for the occurrence of human visceral leishmaniasis in Central-Western Brazil?. Trans R Soc Trop Med Hyg. 2020;114(4):276–283. doi: 10.1093/trstmh/trz110 31851365

[33] Luz JGG, Dias JVL, Carvalho AG, Piza PA, Chávez-Pavoni JH, Bulstra C, Coffeng LE, Fontes CJF. Human visceral leishmaniasis in Central-Western Brazil: Spatial patterns and its correlation with socioeconomic aspects, environmental indices and canine infection. Acta Trop. 2021;221:105965. doi: 10.1016/j.actatropica.2021.105965 34029529

[34] Carvalho AG, Luz JGG, Rodrigues LD, Dias JVLL, Fontes CJF. Impact of socioeconomic status on the knowledge, attitudes, and practices about visceral leishmaniasis among dog owners. J Infect Dev Ctries. 2021 (forthcoming) 10.3855/jidc.14522 34780376

[35] Quinnell RJ, Carson C, Reithinger R, Garcez LM, Courtenay O. Evaluation of rK39 rapid diagnostic tests for canine visceral leishmaniasis: longitudinal study and meta-analysis. PLoS Negl Trop Dis. 2013;7(1):e1992. doi: 10.1371/journal.pntd.0001992 23326615 PMC3542187

[36] Pessoa-E-Silva R, Vaitkevicius-Antão V, de Andrade TAS, Silva ACO, Oliveira GA, Trajano-Silva LAM, et al. The diagnosis of canine visceral leishmaniasis in Brazil: Confronting old problems. Exp Parasitol. 2019;199:9–16. doi: 10.1016/j.exppara.2019.02.012 30796913

[37] Coura-Vital W, Reis AB, Reis LES, Braga SL, Roatt BM, Aguiar-Soares RDO, et al. Canine visceral leishmaniasis: incidence and risk factors for infection in a cohort study in Brazil. Vet Parasitol. 2013;197(3–4):411–417. doi: 10.1016/j.vetpar.2013.07.031 23941965

[38] Jensen LF, Pedersen AF, Andersen B, Fenger-Grøn M, Vedsted P. Distance to screening site and non-participation in screening for breast cancer: a population-based study. J Public Health (Oxf). 2014;36(2):292–299. doi: 10.1093/pubmed/fdt068 23885026

[39] Harada K, Lee S, Shimada H, Lee S, Bae S, Anan Y, et al. Distance to screening site and older adults’ participation in cognitive impairment screening. Geriatr Gerontol Int. 2018;18(1):146–153. doi: 10.1111/ggi.13133 28762614

[40] Amiri S, Pham CD, Amram O, Alcover KC, Oluwoye O, Bravo L, et al. Proximity to screening site, rurality, and neighborhood disadvantage: treatment status among individuals with sexually transmitted infections in Yakima County, Washington. Int J Environ Res Public Health. 2020;17(8):2679. doi: 10.3390/ijerph17082679 32295243 PMC7215758

[41] Barbosa DS. A inserção do Médico Veterinário nos Núcleos de Apoio à Saúde da Família (NASF): novos caminhos de atuação na saúde pública. J Manag Prim Health Care 2014;5(1):1–3. doi: 10.14295/jmphc.v5i1.189

[42] Alvar J, Yactayo S, Bern C. Leishmaniasis and poverty. Trends Parasitol. 2006;22(12):552–557. doi: 10.1016/j.pt.2006.09.004 17023215

[43] Carvalho AG, Luz JGG, Rodrigues LD, Dias JVL, Fontes CJF. Factors associated with *Leishmania* spp. infection in domestic dogs from an emerging area of high endemicity for visceral leishmaniasis in Central-Western Brazil. Res Vet Sci. 2019;125:205–211. doi: 10.1016/j.rvsc.2019.06.013 31260840

[44] Carvalho AG, Alves I, Borges LM, Spessatto LB, Castro LS, Luz JGG. Basic knowledge about visceral leishmaniasis before and after educational intervention among primary health care professionals in Midwestern Brazil. Rev Inst Med Trop Sao Paulo. 2021;63:e56. doi: 10.1590/S1678-9946202163056 34231821 PMC8266306

[45] Coura-Vital W, Marques MJ, Veloso VM, Roatt BM, Aguiar-Soares RD, Reis LE, et al. Prevalence and factors associated with Leishmania infantum infection of dogs from an urban area of Brazil as identified by molecular methods. PLoS Negl Trop Dis. 2011;5(8):e1291. doi: 10.1371/journal.pntd.0001291 21858243 PMC3156685

[46] Harhay MO, Olliaro PL, Costa DL, Costa CH. Urban parasitology: visceral leishmaniasis in Brazil. Trends Parasitol. 2011;27(9):403–409. doi: 10.1016/j.pt.2011.04.001 21596622

[47] Oleson JJ, Brown GD, McCreery R. Essential Statistical Concepts for Research in Speech, Language, and Hearing Sciences. J Speech Lang Hear Res. 2019;62(3):489–497. doi: 10.1044/2018_JSLHR-S-ASTM-18-0239 30950745 PMC6802903

