# Supplementary material for: Passive case detection for canine visceral leishmaniasis control in urban Brazil: Determinants of population uptake
Source: PLoS Negl Trop Dis. 2021 Oct 8;15(10):e0009818. doi: 10.1371/journal.pntd.0009818 (PMC8528332; doi:10.1371/journal.pntd.0009818)
Supplement: S1 Text — This version has been adapted and translated from Portuguese. (DOCX) [file pntd.0009818.s001.docx]

| **Key** | **Variable** | **Response** |
| --- | --- | --- |
| COD | Numeric code |  |
| DATA | Date |  |
| NOME_PROP | Name |  |
| SEXO_PROP | Sex | ( ) Female ( ) Male |
| IDADE_PROP | Age |  |
| RENDA | Family income (Brazilian minimum wages) | ( ) < 1 ( ) 3 --\| 5  ( ) 1 ( ) > 5  ( ) 1 --\| 2 ( ) Do not know  ( ) 2 --\| 3 ( ) Did not answer |
| ESCOLA | Educational level | ( ) Did not study ( ) College (incomplete)  ( ) Primary education ( ) College (complete)  ( ) Elementary education ( ) Postgraduate  ( ) High school (incomplete) ( ) Do not know  ( ) High school (complete) ( ) Did not answer |
| NOME_CAO | Dog’s name |  |
| SEXO_CAO | Dog’s sex | ( ) Female ( ) Male |
| IDADE_CAO | Dog’s age |  |
| TER_CAO | Dog’s finality | ( ) Companion ( ) Guard  ( ) Others:_________________________ |
| EXAME_CAO | Why did you bring the dog to be tested for canine visceral leishmaniasis (CVL)? | ( ) Presence of suggestive CVL signs  ( ) Existence of dogs with CVL in the neighborhood  ( ) Existence of humans with VL in the neighborhood  ( ) Referral from someone else  ( ) Routine / prevention  ( ) Others:__________________________ |
| SINTOMA_CAO | Does the dog have any apparent signs consistent with CVL? | ( ) Yes ( ) No |
| CITE_SINT | If so, what signs? |  |
| TEMPO_SINT | How long has the dog been presenting these signs? |  |
| TESTE_CAO | In case of CVL positivity, what do you intend to do with the dog? | ( ) Isolation  ( ) Treatment  ( ) Euthanasia  ( ) Use of insecticide-impregnated dog collars  ( ) Others____________________________  ( ) Do not know |
| POSITIVO_CAO | It is recommended that a dog with a positive test for CVL should be euthanized. Do you know why? | ( ) Yes ( ) No |
| CITE_POSITIVO | If so, why? |  |
| SACRIFIC_CAO | Would you allow your dog to be euthanized? | ( ) Yes ( ) No  ( ) Not know |
| TRAT_CAO | Do you know if there is treatment for CVL? | ( ) Yes ( ) No |
| TRAT_CAO2 | Have you ever treated your dog for CVL? | ( ) Yes ( ) No  ( ) Not know |
| TRAT_CAO3 | If so, how? |  |
| VACINA_CAO | Do you know if there is a vaccine for CVL? | ( ) Yes ( ) No |
| TRANS | Do you know how VL is transmitted? | ( ) Insect bite ( ) Rat urine  ( ) Contact with dogs ( ) Congenital disease  ( ) Dog biting ( ) Others ____________  ( ) Not know |
| PREV | Do you know how to prevent VL? | ( ) Yes ( ) No |
| CITE_PREV | If so, how? |  |
| OBS | Observations |  |

**QUESTIONNAIRE**
