# Supplementary material for: Passive case detection for canine visceral leishmaniasis control in urban Brazil: Determinants of population uptake
Source: PLoS Negl Trop Dis. 2021 Oct 8;15(10):e0009818. doi: 10.1371/journal.pntd.0009818 (PMC8528332; doi:10.1371/journal.pntd.0009818)
Supplement: S2 Table — Data were extracted from the National Health Survey [19] for the state of Mato Grosso, Brazil. (DOCX) [file pntd.0009818.s003.docx]

**S1 Table:** **Negative binomial regression coefficients for the number of domestic dogs per household in each primary sampling unit as a function of demographic predictors.**

| **Variable** | **Univariate analysis** | | | **Multivariate analysis** | | |
| --- | --- | --- | --- | --- | --- | --- |
|  | **β** | **SE** | ***p*-value** | **β** | **SE** | ***p*-value** |
| Mean number of inhabitants per PPH | 0.180 | 0.077 | 0.019* | 0.113 | 0.075 | 0.131 |
| Mean monthly nominal income per PPH (Brazilian minimum wages)^a^ | -0.144 | 0.077 | 0.063 | - | - | - |
| Proportion of PPHs with literate heads | -0.412 | 0.498 | 0.408 | - | - | - |
| Proportion of PPHs according to the housing type |  |  |  |  |  |  |
| House | 1.083 | 0.240 | <0.001* | 1.006 | 0.239 | <0.001* |
| Apartment | -1.080 | 0.245 | <0.001* | - | - | - |
| Proportion of PPHs with public water supply | 0.047 | 0.240 | 0.845 | - | - | - |
| Proportion of PPHs with public garbage collection | -1.365 | 0.607 | 0.025* | - | - | - |
| Proportion of PPHs with sewage system | -0.142 | 0.107 | 0.185 | - | - | - |
| Constant | - | - | - | -1.325 | 0.289 | <0.001* |

Data were extracted from the National Health Survey (2013) for the state of Mato Grosso, Brazil.

SE: standard error. PPH: permanent private households.

^a^ Brazilian minimum wage (2013) = US$ 154.4 (R$ 678).
