# Supplementary figures and images for: Passive case detection for canine visceral leishmaniasis control in urban Brazil: Determinants of population uptake
Source: PLoS Negl Trop Dis. 2021 Oct 8;15(10):e0009818. doi: 10.1371/journal.pntd.0009818 (PMC8528332; doi:10.1371/journal.pntd.0009818)

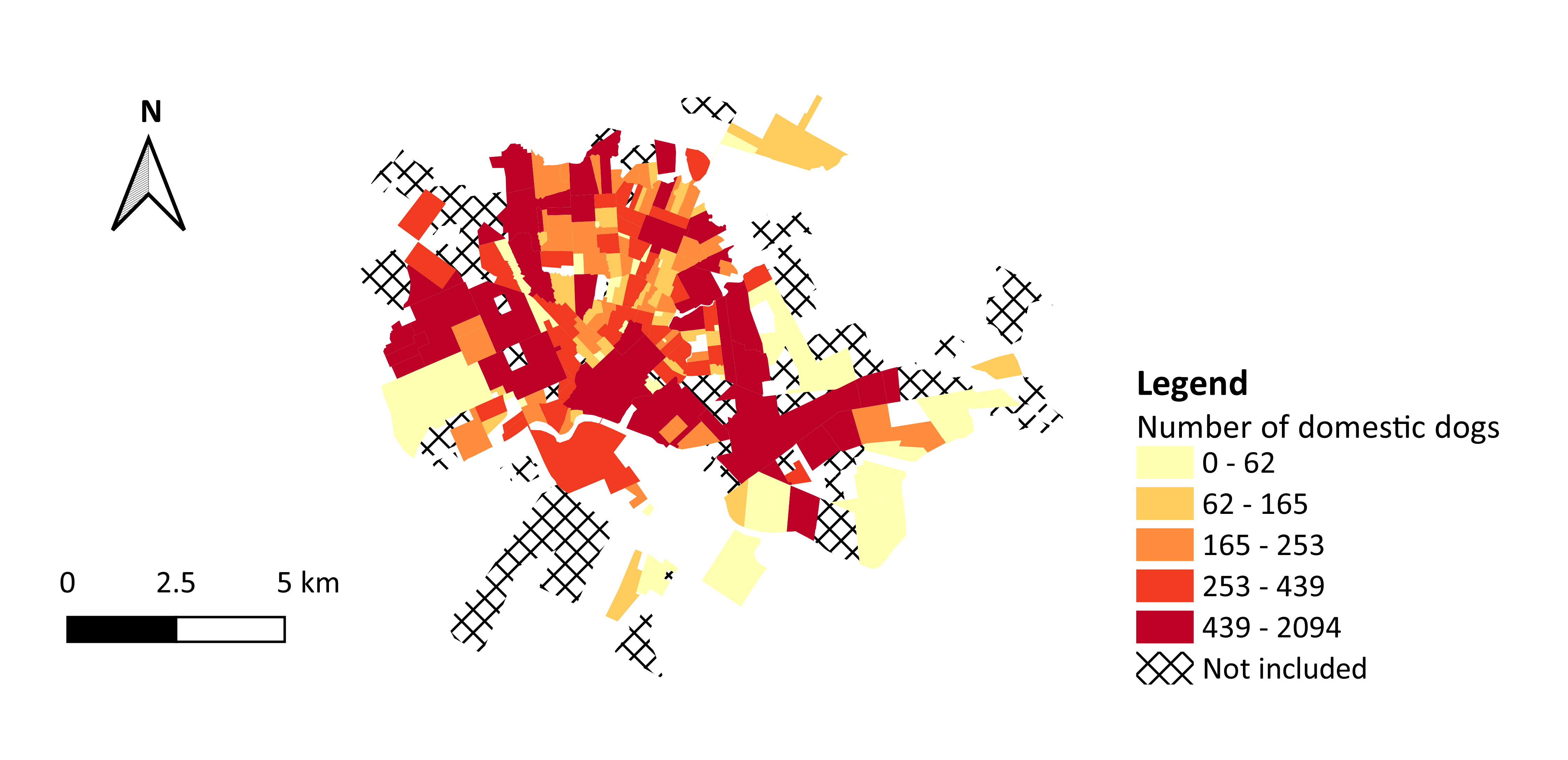

Supplement: S1 Fig — In the legend, colors represent ranges of neighborhood-level values, where the upper bounds are included within interval. Digital georeferenced database of the neighborhoods was provided by the Municipal Health Department of Rondonópolis. (TIF) [file pntd.0009818.s004.tif]
